# The effect of antifungal extracts on the contamination of grain with microfungi

**DOI:** 10.1002/fsn3.1384

**Published:** 2020-02-05

**Authors:** Ilona Keriene, Audrone Mankeviciene, Jaune Blazyte

**Affiliations:** ^1^ Šiauliai University Šiauliai Lithuania; ^2^ Lithuanian Research Centre for Agriculture and Forestry Akademija Lithuania

**Keywords:** antifungal activity, bee product, buckwheat, extracts, grain, microfungi

## Abstract

The study aimed to analyze the effects of extracts made from buckwheat grain, hulls, and bee products (propolis, bread, and pollen) and extraction solvents on the growth of microfungi on a medium and on buckwheat, wheat, oat, and maize grain. Research findings suggest that bioactive compounds contained in buckwheat grain reduced the amount of *Fusarium* spp. in the grain kept in the antifungal extract for 90 min at 25°C temperature. Buckwheat hull extract was more effective in inhibiting mycelial growth of mycotoxin‐producing *Fusarium culmorum* and *Fusarium graminearum* compared with buckwheat grain extract (13%–50% and 14%–36%, respectively). The antifungal activity of extracts of bee products did not depend on the content of phenolic compounds in them; however, it depended on the grain species treated. After treatment of oat, wheat, and maize grain with bee product extracts, the lowest concentration of microfungi was identified on oat grain. More significant analysis results were obtained for the samples where ethanol solvent had been used for the preparation of extracts.

## Graphical abstract

This paper focusses on the application of natural products for the protection of food raw materials against microfungal contamination. It is not possible to completely prevent grain contamination, because the reduction of the amount of predominant microfungi on grain creates conditions for the reproduction of propagules of other fungi. It is very important that the antifungal activity of bioactive compounds should be targeted at mycotoxin‐producing genera and species of fungi.

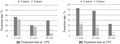

## 1 INTRODUCTION

The United Nation's Food and Agriculture Organization (FAO, 2008) points out that the contamination of food raw materials and products with bio‐contaminants is increasing and one of the most important problems of everyday life is the negative impact of mycotoxin‐producing microfungi on food quality. Laca, Mousia, Díaz, Webb, and Pandiella (2006) indicate that the types of microorganisms present in the wheat grain surface are not necessarily the same as those found in inner layers. The majority of the bacteria and the microfungi of wheat grains are strongly adhered to the grain and are located in the pericarp that surrounds the endosperm and the germ (Laca et al., 2006). This facilitates transmission of microfungi further along the food chain. Studies by Tournas and Niazi (2018) found that wheat whole grain flour was contaminated with *Aspergillus* spp. and *Fusarium* spp. Microfungi may contaminate fresh, minimally processed vegetables or sprouts. The most frequently isolated molds from sprouts purchased from local supermarkets were *Alternaria*, *Cladosporium*, *Penicillium*, and *Phoma* (Tournas, 2005). Therefore, reducing the loads of microfungi on foods is very important because it often reduces grain or food quality and can potentially produce mycotoxin. It is impossible to completely prevent microfungal contamination of food raw materials (Bullerman & Bianchini, 2007); therefore, researchers are searching for natural ways to increase food safety and extend product shelf‐life. Research shows that bioactive compounds present in plant‐derived food raw materials and products can help protect food raw materials against various causal agents; therefore, their extracts can be used as natural fungicides (Ansari, Anurag, Fatima, & Hameed, 2013). Lukšienė et al. (2007) have found that 5‐aminolevulinic acid is a highly effective antifungal biological compound, which stimulates the growth of wheat seedlings and roots and induces the augmentation of chlorophyll content. A large proportion of bioactive compounds are phenolic compounds, which, depending on the chemical structure and properties, can penetrate the cell wall of a microscopic fungus and affect mycelial development, and disrupt the synthesis of proteins and mycotoxins (Ansari et al., 2013; Pagnussatt et al., 2013).

Phenolic compounds soluble in a cell cytosol make up a large proportion of biologically active substances in buckwheat, and there are significantly less cell‐wall bound phenolic acids (Guo et al., 2011; Li, Yuan, Yang, Tao, & Ming, 2013). Compared to other crops, buckwheat is less susceptible to diseases (Lugauskas, Krasauskas, & Repečkienė, 2004), its extracts are characterized by antifungal properties (Lattanzio, Lattanzio, & Cardinali, 2006) and antimicrobial activity (Čabarkapa, Sedej, Sakač, Šarić, & Plavšić, 2008); however, our previous research has shown that under favorable environmental conditions buckwheat grains can be contaminated with microfungi and mycotoxins synthesized by them (Keriene, Mankeviciene, & Cesnuleviciene, 2018). Therefore, in order to protect the grain from the effects of toxic microfungi, it is important to identify relevant conditions for their prevention.

Bee propolis, bee bread, and bee pollen are also rich in bioactive compounds, phenolic compounds in them are mostly in the form of flavonoids and their concentration depends on the plant species, growing season and the habitat (Ivanišová et al., 2015; Rzepecka‐Stojko et al., 2015; Viuda‐Martos, Ruiz‐Navajas, Fernández‐López, & Pérez‐Álvarez, 2008). More than 300 compounds have been identified in propolis, 5% of which are polyphenols, esters, terpenes, beta‐steroids, aromatic aldehydes, and alcohols, stilbenes (Viuda‐Martos et al., 2008). Various techniques for extraction, separation, identification, and quantification of phenolic compounds have been developed to capitalize and characterize biologically active constituents from bee products (Spulber, Colța, Băbeanu, & Popa, 2017; Spulber, Doğaroğlu, Băbeanu, & Popa, 2017). The research shows that extraction procedures applied for phenolic compounds do not present significant differences (Carpes, Begnini, Alencar, & Masson, 2007; Spulber, Colța, et al., 2017; Spulber, Doğaroğlu, et al., 2017).

Bee products are known for their antibacterial properties (Ivanišová et al., 2015; Kujumgiev et al., 1999) often used as inhibitors of *Candida* genus (Gucwa, Kusznierewicz, Milewski, Dijck, & Szweda, 2018); however, there is a paucity of scientific information on their use in preventing grain contamination.

The present study aimed to estimate the effect of extracts produced from buckwheat grain, hulls, and bee products (bee propolis, bee bread, and bee pollen) in reducing buckwheat, spring wheat, oat, and maize grain contamination with pathogenic microfungi.

## 2 MATERIALS AND METHODS

### 2.1 Samples

The samples of buckwheat grain, from which grain and hull extracts were produced, as well as the samples of spring wheat, oat, and maize grain were collected in the commercial fields of Lithuania in the 2016 and 2017 seasons.

### 2.2 Origin of fungal material

The species of *Fusarium graminearum* and *Fusarium culmorum* were isolated from wheat samples collected at Lithuanian Research Centre for Agriculture and Forestry. Mycelial plugs were obtained from 7‐day‐old *Fusarium* cultures grown on a PDA using a sterile cork borer. The plugs were placed in the center of a fresh plate with the mycelial side facing the agar.

### 2.3 Preparation of antifungal extracts from buckwheat and bee products

All the chemicals were of analytical grade and were used as received. Samples were milled in an IKA A11 Basic mill (Staufen, Germany) and stored at +4°C until analysis. Bioactive compounds from buckwheat grain and hull samples (2.500 ± 0.001 g) were extracted with 75% (v/v) aqueous methanol (25.0 ± 0.1 ml) at room (21 ± 1°C) temperature for 15 hr in an orbital shaker Heidolph Vibramax under constant shaking. The mixtures were centrifuged (Hermle, Germany) for 10 min at 4,000 rpm.

The bee bread and bee pollen extracts were prepared in the same way as buckwheat grain extract, but by using 25.0 ± 0.1 ml 70% ethanol instead of methanol or undiluted DMSO solvent. The propolis extract was prepared from 0.250 ± 0.001 g of crushed propolis in 25.0 ± 0.1 ml 70% ethanol or DMSO solvent. Extraction was performed for 5 hr at 150 rpm.

### 2.4 Determination of total phenolic content in buckwheat and bee products

Total phenolic content (TPC) was determined using a Folin–Ciocalteu reagent (Kerienė et al., 2015): TPC was analyzed by mixing 7.9 ml of deionized water, 100 μl extract, 0.5 ml Folin–Ciocalteu, and 1.50 ml 20% sodium carbonate (after 6 min at room temperature). The absorbance (after 120 min) was measured at 760 nm using a UV/VIS spectrophotometer Genesys 10‐UV. A standard curve (0.05–1.5 mg/ml) was prepared with rutin. The final results were expressed as mg of rutin eq/g dry weight (DW).

### 2.5 Determination of grain contamination with microfungi

An agar plate method was used for grain infection estimation. Grains were plated in Petri dishes with a potato dextrose agar (PDA), supplemented with 20% citric acid additive and incubated for 7–14 days at 23 ± 2°C in the dark (Mathur & Kongsdal, 2003). The morphological identification of microfungi was carried out using an optical microscope (Nicon Eclipse E 200). Fungal colonies were identified, and the contamination percentage was estimated according to the number of contaminated grains. The overgrown *Fusarium* spp. colonies were isolated, purified, and identified according to the manuals of Nelson, Toussoun, and Marasas (1983) and Leslie and Summerell (2006). Grain infection with other fungal species (*Aspergillus* spp., *Penicillium* spp.) was estimated and identified according to the manual of Sutton, Fothergill, and Rinaldi (2001).

### 2.6 Buckwheat grain treatment with antifungal extract

Buckwheat grains (5 g) were treated with 1 ml of raw extracts isolated from other buckwheat grain. Different exposure times (15, 45, 90 min) and temperatures (18°C, 25°C) were used. The control treatment was sterile H_2_O and 75% methanol diluted with 1:4 sterile H_2_O. The treated grains were plated in Petri dishes with PDA, supplemented with 20% citric acid and incubated for 7 days at 23 ± 2°C in the dark.

### 2.7 The growth of *Fusarium* species on a PDA medium treated with antifungal extracts

The antifungal activity of buckwheat grain and hull extracts in controlling mycotoxin‐producing *Fusarium* species was evaluated in an in vitro assay. The buckwheat grain and hull extracts used for the assessment of growth inhibition of *F. culmorum* fungi were produced in ethanol while those used for growth inhibition of *F. graminearum* fungi were produced in a dimethyl sulfoxide (DMSO) solvent. The assay was conducted according to Hussin et al. (2009) methodology with small modifications. Sterile Petri dishes were filled with sterile potato dextrose agar (PDA), supplemented with 20% citric acid. The medium was cooled down to 40°C, the plates were filled with 100 µl of undiluted buckwheat extract and hull extract diluted twice with a DMSO solvent. Next, the center of each Petri dish was inoculated with 5 mm diameter fragment of fungal mycelium. The inoculated dishes were incubated for 7 days in a thermostat (Binder GmbH) at 23 ± 1°C. The mycelial growth was measured periodically every 24 hr. Ethanol and DMSO solvents were used as control.

### 2.8 Wheat, oats, and maize grain treatment with extracts of bee products

Grains of spring wheat, oats, and maize were plated in Petri dishes, 100 grains per dish. Then, they were treated with 5 g: 1 ml of twice diluted with distilled water extracts of bee products prepared in ethanol and DMSO solvents. The control treatment was 70% ethanol and DMSO solvent. After treatment, the grains were left for 1 hr to dry and for the solvent to evaporate. Then, the treated grains were placed into Petri dishes, 10 grains per dish, on a PDA medium in not less than 8 replications and incubated in a thermostat at 23 ± 1°C. The variation of microscopic fungi content was estimated after 7 days at 23 ± 1°C in the dark.

### 2.9 Statistical analysis

Analyses of phenolic compounds in the samples were repeated twice and expressed as a mean ± standard deviation (*SD*) of Microsoft Office Excel 2016 (“Microsoft”, USA). Statistical analysis was done by using packages from the software *SAS® Enterprise Guide 7.1*—one way *ANOVA.* Significant differences in the antifungal activity of extracts were estimated using Fisher's least significant difference tests. Results with values *p ≤ .05 and **p ≤ .01 were considered significant.

## 3 RESULTS AND DISCUSSION

### 3.1 Total phenolic content in antifungal extracts

The highest content of phenolic compounds was identified in the propolis extract (15.5 mg/g d.w.), which was nearly twice as high as in the bee bread extract and 20% higher than in the bee pollen extract. The content of phenolic compounds in buckwheat hulls was 16% higher than in grain (Table 1). This shows that with dehulling part of phenolic compounds is lost. Different solvents used for the preparation of bee product extracts did not have significant effect, therefore, in Table 1 we presented average TPCs determined in ethanol and DMSO solvents.

**Table 1:** Total phenolic content in antifungal extracts

Extracts	Buckwheat products		Bee products		
	Grain	Hulls	Pollen	Bread	Propolis
Total phenolic content, mg/g dry weight	9.0 ± 0.4	12.8 ± 2.1	9.9 ± 0.5	8.3 ± 1.3	15.5 ± 0.5

Our results agree with those of Spulber, Colța, et al. (2017); Spulber, Doğaroğlu, et al. (2017), who suggest that propolis and bee pollen are considered the main bee products with the highest amounts of phenolic compounds and different solvent applied for bioactive substances extraction from bee products do not present significant differences. Our previous studies have shown that buckwheat grain and hulls have the highest amount of total phenolic compounds with the highest antioxidant activity, compared with other cereal grain **(**Kerienė et al., 2015). The content of individual phenolic compounds in the total phenolics in buckwheat grain and other parts of grain and plant depends on the soil type, cultivation region, and microclimate (Guo et al., 2011; Zielińska, Turemko, Kwiatkowski, & Zieliński, 2012).

### 3.2 Microscopic fungi in untreated cereal grain

Natural grain contamination with microfungi was estimated before treatment with the antifungal extracts. All buckwheat grain samples tested positive for microscopic fungi, with the fungi of *Fusarium* genus being dominant (Table 2), and mycotoxin‐producing *F. graminearum* species was identified (one *F. graminearum* species). Stored wheat, oat, and maize grains were mostly infected with storage fungi. The fungi of *Penicillium* genus were dominant on wheat and maize grain, while *Aspergillus* fungi were prevalent on oats (Table 2). Contamination with these fungi is detrimental because they cause plant diseases, produce toxic mycotoxins and cause allergic reactions in humans (Baxi et al., 2016).

**Table 2:** The main genera of microfungi dominant on untreated grain

Sample	Buckwheat	Wheat	Oats	Corn
Dominant fungi genera	*Fusarium* spp.	*Penicillium* spp.	*Aspergillus* spp.	*Penicillium* spp.
Contamination %	80	100	48	88
Other microfungi %	20	0	23	8
Noncontaminated %	0	0	31	4

The next stage of the current study involved analysis of antifungal properties of bee products and buckwheat grain and hulls extracts. We aimed to ascertain whether bioactive compounds present in buckwheat and bee products can help reduce contamination of food grains with harmful microfungi.

### 3.3 Impact of buckwheat grain and hulls extracts on the growth of *Fusarium s*pp

**Figure 1:**
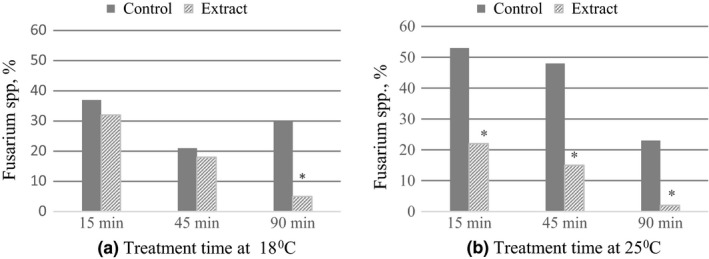
The influence of treatment time and temperature of antifungal extract on *Fusarium* spp. variation in buckwheat grain. *Significant at *p* ≤ .05

The study showed that buckwheat grain extract has an antifungal effect on *Fusarium* spp. fungi, but inhibition of their growth was influenced by the incubation temperature and duration of grain treatment. The highest *Fusarium* spp. fungi suppression in vitro was revealed with buckwheat extract at 18°C and 25°C incubation temperature and exposure time of 90 min: *Fusarium* spp. significantly decreased by 71% and 84% (*p* ≤ .05), compared with the control (Figure 1). Significantly less (38%) *Fusarium* spp. fungi on grain were detected at extract exposure times of 15 and 45 min and incubation temperature of 25°C. However, at the same exposure times but reduced incubation temperature to 18°C, the differences in *Fusarium* spp. fungi content were insignificant, compared with the control.

The results of other researchers suggest that the duration of contact between the bioactive compounds and the pathogen is an important factor in assessing the efficacy of the antifungal extract. Rahman, Al‐Reza, Siddiqui, Chang, and Kang (2014) have reported that antifungal activity of *Lonicera japonica* essential oil against dermatophyte *Microsporum canis* KCTC 6348 was observed with the increase in exposure time from 30 to 150 min. At low concentrations of extracts, significant rate of inhibition was the characteristic feature of the essential oil.

Methanol solvent had inhibitory effect on grain contamination with *Fusarium* spp. fungi. Incubation of grain at 18°C in 70% methanol solvent for 15 min and 45 min reduced *Fusarium* spp. fungi on grain by 2–4 times, compared with the untreated grain. At an incubation temperature of 25°C, the influence of methanol solvent on the growth of fungi was weaker, and the effect of extracts was more pronounced (Figure 1). The control treatment was simultaneously used in which buckwheat grain was treated with sterile distilled water. Results indicated that irrespective of the incubation temperature and exposure time the contamination of grain with *Fusarium* spp. fungi treated with only distilled water varied little and was similar to that before treatment 70%–88%.

In the course of this study, a tendency was revealed showing that a decrease in *Fusarium* spp. fungi growth created favorable conditions for the spread of other fungi, and their diversity depended on the incubation temperature of the extracts used and the duration of grain exposure to the extracts. When incubating at 18°C, the fungi of the genus *Alternaria* began to dominate on the buckwheat grains kept in the extracts for 15 min. When keeping the grain in the extracts for 90 min, contamination with fungi of the genus *Penicillium* increased, and the mycelia of the *Fusarium* and *Alternaria* genera were altered and poorly developed. Having increased incubation temperature to 25°C, the content of *Alternaria* spp. fungi on the grain kept in the extracts for 90 min was low; however, contamination with the fungi of *Penicillium* genus made up nearly 30%. It is likely that with a reduction in the dominating fungi of *Fusarium* genus on buckwheat grain surface, contamination with the fungi of other genera (*Alternaria*, *Penicillium*) increased due to lesser competition. Natural conditions for grain contamination with *Fusarium* and *Alternaria* fungi are similar (Los, Ziuzina, & Bourke, 2018), but *Fusarium* spp. fungi grow faster and need less nutrients for development than *Alternaria* spp. (Weikl, Ghirardo, Schnitzler, & Pritsch, 2016), therefore, the reduction in *Fusarium* spp. fungi favoured conditions for the growth of *Alternaria* fungi. An increased temperature to 25°C favoured development of propagules of *Penicillium* fungi, as *Penicillium* maximum growth in vitro is obtained at 23°C (pH 3–4.5) (Public health expertise & reference centre, 2016). Our study revealed a trend showing that bioactive compounds in buckwheat grain have greater effect in inhibiting growth of fungi occurring under field conditions (*Fusarium*, *Alternaria* genera); however, they have little impact of the growth of *Penicillium* fungi. These findings complement the results obtained in our previous study, which suggests that buckwheat grain phenolic compounds were found to decrease the risk of *Fusarium‐produced* mycotoxin occurrence in grain: with increasing concentrations of rutin, quercetin, and total phenolics content in buckwheat hulls and grain samples, the contents of trichothecene mycotoxins (deoxynivalenol, T‐2 toxin) were significantly (*p* ≤ .05) lower (Keriene et al., 2018).

**Figure 2:**
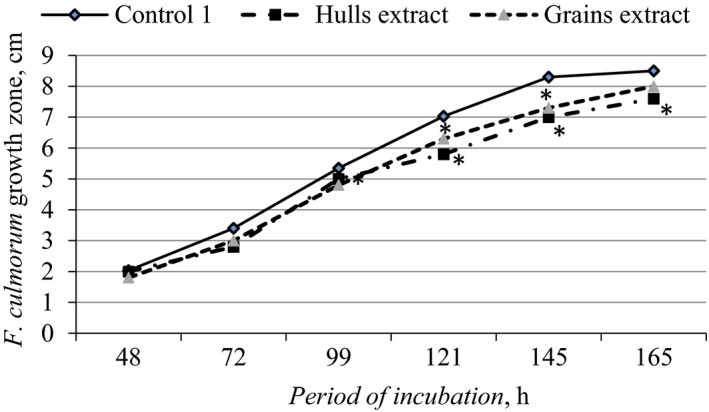
The antifungal activity of buckwheat hull and grain extracts in ethanol against *Fusarium culmorum*, Control 1—75% ethanol solvent; *—significant differences, *p* ≤ .05

In order to estimate the effects of buckwheat grain and hull extracts on the growth of mycotoxin‐producing *F. culmorum* and *F. graminearum* species on buckwheat grain, in vitro tests were done in which *F. culmorum* and *F. graminearum* were cultivated on the PDA media supplemented with buckwheat grain and hull extracts. The data of measurement of growth of mycelia showed that significant antifungal activity evidence (*p* ≤ .05) of buckwheat hull extract for mycelial growth of *Fusarium* monoculture was determined 96 hr after supplementation of PDA with the extract (Figure 2). During this test period and up to 165 hr, the growth of *F. culmorum* mycelium was 0.2–1.3 cm smaller, and that of *F. graminearum* 0.6–1.8 cm smaller, compared with the control treatments. The strongest antifungal activity of hull extract was determined after 165 hr of incubation.

The antifungal effect of buckwheat grain extract on *F. culmorum* growth was from 13% to 50% and on *F. graminearum* growth from 14% to 29% weaker compared with that of hull extracts. Significant (*p* ≤ .05) antifungal activity evidence of grain extract on *F. culmorum* and *F. graminearum* growth was determined during one measurement period after 121 hr to 145 hr of incubation (Figure 2). Later the impact of grain extract on the growth of microfungi monocultures was insignificant.

The antifungal activity of individual phenolic compounds on the growth of *Fusarium* species is related to their chemical properties and concentration (Pani et al., 2016; Schöneberg et al., 2018). It is likely that these reasons determined higher antifungal effect of hull extracts compared with grain extract, as our previous research has shown that hulls have higher contents of phenolic compounds and their diversity is different (Kerienė et al., 2015). This is important for the grain since hull can protect the embryo from mycotoxins produced by microfungi. Pani et al. (2016) suggest that having treated *F. culmorum* with 8 phenolic compounds, their effect on mycelial growth and concentration of deoxynivalenol synthesized by this fungus was different: from maximally effective fungicidal effect (magnolol) to 116.57 dry fungal biomass relative yield (Me‐dehydrozingerone). According to Schöneberg et al. (2018), *F. graminearum* mycelial growth is influenced by the concentration of phenolic compounds and conditions can be created which would not inhibit but promote mycelial growth.

To ensure the reliability of the research results, we estimated the effect of solvents on the growth of *Fusarium* species. It was found that *F. culmorum* monoculture is sensitive to 70% methanol solvent, while *F. graminearum* growth was inhibited by 70% ethanol. DMSO solvent had the weakest effect on the growth of *F. graminearum* monoculture.

### 3.4 Impact of extracts of bee products on the growth of microscopic fungi on wheat, oat, and maize grain

**Figure 3:**
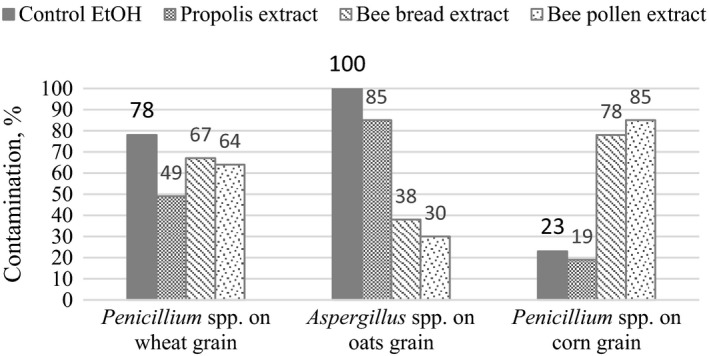
Grain contamination with microscopic fungi after treatment with extracts of bee products. Note. EtOH—75% ethanol solvent

In another stage of our study, we analyzed the antifungal activity of bee product extracts against microfungi on spring wheat, oat, and maize grain. The findings showed that bee product extracts prepared in 75% ethanol and DMSO solvents inhibited the growth of *Penicillium* spp. fungi on wheat grain (Table 2). *Penicillium* spp. fungal contamination on wheat grains treated with propolis extract was 30% lower, of those treated with bee pollen extract was 14% lower and of those treated with bee bread extract was 11% lower, compared with the control (Figure 3). Extracts prepared in a DMSO solvent inhibited the growth of *Penicillium* spp. on wheat grain by 32% (bee bread extract) to 19% (bee pollen extract), compared with the control (Figure 3).

Untreated maize grains were also heavily contaminated with *Penicillium* spp. fungi (Table 2); however, bee bread and bee pollen ethanolic extracts had little effect—the level of contamination after treatment remained very similar to that before treatment. Propolis extract inhibited the growth of *Penicillium* spp., ethanol solvent also had effect on the growth of these fungi. The incidence of *Penicillium* spp. fungi was low after treatment with propolis extract; however, ethanol solvent also had effect on the growth of these fungi. The study indicated that the total contamination of maize grain with microfungi did not decrease, as other fungal species started to dominate (Table 3). Extracts of bee products prepared in the DMSO solvent did not have significant effect on the growth of *Penicillium* spp. fungi on maize grain.

**Table 3:** The average number of microfungi on grain

Extracts	Wheat grain		Oats grain		Corn grain	
	Average^a^ ± *SD*	*p* value	Average ± *SD*	*p* value	Average ± *SD*	*p* value
Control^b^	8.5 ± 1.7		10.0 ± 0.3		9.9 ± 2.3	
Propolis	6.4 ± 2.3*	.03	8.7 ± 1.1**	.01	8.7 ± 1.6	.19
Bee bread	8.0 ± 1.1	.45	6.5 ± 1.3**	<.01	9.7 ± 2.3	.84
Bee pollen	7.3 ± 2.2	.19	7.8 ± 2.4**	.01	10.1 ± 0.3	.78

Note

*p* value—confidence interval.

a Average number of microfungi per petri dish (10 replications).

b 75% ethanol solvent.

* Significant differences at *p* ≤ .05.

** Significant differences at *p* ≤ .01.

*Aspergillus* spp. fungi dominating on oat grain were significantly influenced by ethanolic bee pollen and bee bread extracts; however, ethanolic extract of propolis had hardly any inhibiting effect on the growth of these fungi (Figure 3). Bee product extracts prepared in the DMSO solvent did not have significant negative effect on the growth of *Aspergillus* spp. fungi.

The study showed that because of the reduction in the content of dominant fungi on the grains treated with the extracts and in the control treatments with ethanol and DMSO, the incidence of other genera of microfungi on the grain was identified. *Aspergillus* spp., started to dominate on wheat grain, the content of these fungi on oat grain increased even more, compared with the contamination level before treatment; therefore, the total grain contamination level in some treatments remained high after treatment.

All ethanolic extracts of bee products significantly inhibited the growth of microfungi on oat grain, while only ethanolic extract of propolis had significant effect on wheat grain contamination (Table 3). Extracts of bee products prepared in the DMSO solvent did not exhibit any antifungal activity, even the opposite results were obtained: on wheat grain treated with bee pollen extracts, the content of microfungi was 9% significantly (*p* ≤ .05) higher than on the grain in the control treatment (Table 3).

The findings of the study showed that propolis extract had the highest content of phenolic compounds (Table 1); however, no significant differences in antifungal activity were determined between the extracts of the bee products. The antifungal activity of the extracts was more dependent on the treated grain species. A trend was revealed showing that propolis extracts were more effective at inhibiting the growth of microfungi on wheat and maize grain, whereas bee bread extract gave better inhibition of microfungi on oat grain.

In this assay ethanol had influence on the growth of microfungi on grain; however, it is likely that synergetically acting together with the bioactive compounds of the extracts ethanol more effectively inhibited the growth of microfungi and therefore it is a more suitable solvent for the preparation of antifungal extracts compared with DMSO.

Scientific literature states that the toxicity of solvents to test organisms or seed germination depends on their type and concentration (Eloff, Masoko, & Picard, 2007; Stutte, Eraso, Anderson, & Hickey, 2006). Ethanol has been known as a good solvent for polyphenol extraction and is safe for human consumption. DMSO solvent is used as a nutritional supplement in medicine (Brien, Prescott, Bashir, Lewith, & Lewith, 2008). Methanol solvent is not suitable for contact with food, but is convenient for evaluating efficacy of other solvents. Moreover, Stutte et al. (2006) suggest that the use of exposure guidelines for humans is not applicable to plant systems: radish sprouts were more tolerant of 285 ppm concentration of methanol compared with 100 ppm concentration of ethanol.

The polarity of solvents is important for the extraction of bioactive compounds; therefore, the same extraction solvent can extract different composition and amount of bioactive compounds (Eloff et al., 2007; Kaur, Kalia, Kumar, & Harjai, 2013). Research has shown that the maximum constituents were extracted from bee pollen with water as a solvent and from propolis with ethanol. Results revealed that 75% ethanol/water solvent may be the best for the highest extraction yield of phenolic compounds of propolis extracts (Kaur et al., 2013; Sun, Wu, Wang, & Zhang, 2015). Our study showed that there was no difference between ethanol and DMSO solvents used for the extraction of phenolic compounds from bee products. However, according to Burdejova and Polovka (2017), 50% ethanol is the most suitable extraction system for the extraction of total phenolic compounds from medicinal plants. Their concentration decreased in the following order: 50% ethanol > distilled water > DMSO.

In conclusion, the antifungal properties of the extracts manifested themselves best when grains had been exposed to them for the longest time—*Fusarium* spp. growth on buckwheat grain was best inhibited when the exposure time was 90 min at 25°C temperature. Hulls are an important part of buckwheat grains, and the bioactive compounds contained in them were more effective in inhibiting the growth of mycotoxin‐producing fungi compared with buckwheat grain extract: *F. culmorum* from 13% to 50% and *F. graminearum* from 14% to 29%.

The antifungal activity of extracts produced from bee products did not depend on the content of phenolic compounds in them but was related to the grain species. Extracts of bee pollen, bee bread, and propolis significantly (*p* ≤ .05) reduced the content of microfungi on oat grain, compared with the control. Our findings suggest that extracts of bee products produced by using ethanol solvent exhibited better antifungal activity compared with the extracts produced using a DMSO solvent.

From a practical point of view, environmentally friendly extracts can be used as an inexpensive preventive measure, for example, to reduce seed contamination with microfungi and mycotoxins before sprouting. However, it should be noted that fungal contamination of grain cannot be completely eliminated, as inhibition of growth of the dominant microscopic fungi by the bioactive compounds creates conditions for the proliferation of propagules of other fungi. Therefore, it is very important that antifungal effect is specifically targeted at the genera and species of mycotoxin‐producing fungi.

## CONFLICT OF INTEREST

The authors declare that they do not have any conflict of interest.

## ETHICAL APPROVAL

This study does not involve any human or animal testing.
